# Remote work transition amidst COVID-19: Impacts on presenteeism, absenteeism, and worker well-being—A scoping review

**DOI:** 10.1371/journal.pone.0307087

**Published:** 2024-07-18

**Authors:** Behdin Nowrouzi-Kia, Alexia M. Haritos, Bao-Zhu Stephanie Long, Chantal Atikian, Luke A. Fiorini, Basem Gohar, Aaron Howe, Yiyan Li, Ali Bani-Fatemi

**Affiliations:** 1 Department of Occupational Science and Occupational Therapy, Temerty Faculty of Medicine, University of Toronto, Toronto, Ontario, Canada; 2 Krembil Research Institute, University Health Network, Toronto, Ontario, Canada; 3 Centre for Research in Occupational Safety & Health, Laurentian University, Sudbury, Ontario, Canada; 4 Centre for Labour Studies, University of Malta, Msida, Malta; 5 Department of Population Medicine, University of Guelph, Guelph, Ontario, Canada; Universiti Pertahanan Nasional Malaysia, MALAYSIA

## Abstract

**Background:**

The COVID-19 pandemic has accelerated the transition to remote work, leading to increased attention on presenteeism and absenteeism among remote workers. Understanding the implications of these phenomena on worker health and productivity is crucial for optimizing remote work arrangements and developing policies to improve employee well-being.

**Objectives:**

This scoping review aims to examine the occurrence of presenteeism and absenteeism among remote workers during the COVID-19 pandemic and the interrelated physical and mental health issues during these periods.

**Methods:**

PsycINFO, Medline, Embase, CINAHL, Eric, Business Source Premier, SCOPUS, and sociological abstracts were searched resulting in 1792 articles. Articles were included if the population of interest was 18+ (i.e., working age), engaged in full or part-time work, and the employees shifted from in-person to remote work due to the COVID-19 pandemic. All study designs, geographical areas, and papers written post-onset of the COVID-19 pandemic were included; however, systematic reviews were excluded. Data was charted into Microsoft Excel by 2 independent reviewers.

**Results:**

The literature search identified 10 studies (i.e., seven cross-sectional studies, two qualitative studies, and one observational study). Five major overarching themes were identified specifically (1) telework and mental health (2) telework and physical health (3) worker benefits (4) gender dynamics and (5) difficulty navigating the teleworking environment. While remote work offers flexibility in terms of saved commute time and flexible work schedules, it also exacerbates challenges related to presenteeism, absenteeism, and work-life balance. These challenges include experiencing psychological distress, depression, anxiety, stress, sleep deprivation, musculoskeletal pain, difficulties concentrating at work for both women and working parents, struggles disconnecting after hours, and the inability to delineate between the work and home environment.

**Discussion:**

The findings suggest that remote work during the COVID-19 pandemic has both positive and negative implications for worker well-being and productivity. However, future research needs to incorporate the potential effects of telework frequency (full time vs. part time) on employee productivity and its role on presenteeism and absenteeism, to gain a more comprehensive understanding on remote work difficulties. Addressing these challenges requires proactive interventions and support mechanisms to promote worker health and productivity in remote settings.

## Introduction

The COVID-19 pandemic prompted widespread shifts to remote work among businesses worldwide, driven by the need to adhere to public health measures while maintaining productivity [1]. Remote work enabled workers to maintain productivity while adhering to safety guidelines and mitigating the spread of COVID-19. The new virtual environment, however, has brought about many repercussions that have affected the physical (e.g., musculoskeletal pain [2]) and mental health of workers (e.g., depression [3]). These consequences, as observed in empirical research studies [4, 5], encompass the various impacts of remote work most noticeably with heightened stress levels faced by employees who engage in work despite illness [6].

Presenteeism, characterized by working while ill or unable to perform work tasks to full capacity, can lead to reduced work productivity and negative health outcomes [7]. On the other hand, absenteeism involves an employee’s failure to attend work according to their schedule [8]. Remote work complicates these concepts, as teleworkers may work while ill from home, attending work virtually or not attending at all [9, 10].

Absenteeism not only entails one’s physical absence from work but also the failure of the employee to perform their remote work [11]. This blurring of boundaries amplifies various issues, including but not limited to reduced work productivity and financial losses for their employer [12]. In addition, sickness presenteeism is said to be more costly than absenteeism as it hinders organizational abilities to remain productive during the time of the pandemic [13].

Up to 69% of remote workers have reported missing work due to illness, indicating the need to address health challenges in remote work environments [14]. While businesses have increased their telecommunication infrastructure to support remote work from home [15] organizations face difficulties in managing presenteeism and absenteeism in remote work settings [16]. This may exacerbate social disparities and affect health and productivity [16].

Transitioning to remote work necessitates adjustments to non-traditional settings, potentially leading to distractions and decreased productivity [16]. Despite these challenges, there is an expectation for workers to maintain pre-pandemic productivity levels [17]. This pressure is compounded by employers’ use of time tracking systems, may contribute to overworking, neglect of work-life balance, and burnout symptoms [18–20]. This continuous pressure to remain constantly available at work can lead employees to develop misconstrued beliefs about their attendance, overlook the importance of taking breaks, self-care, and managing their own health, resulting in presenteeism [21].

The emphasis on occupational health and productivity has heightened due to the increased expectation for employees to seamlessly adapt to remote work [22]. While working from home is often seen as more convenient, individuals may find themselves working in suboptimal conditions that would not be typically tolerated in an office setting. In-person work environments may encourage staff to take time off to prevent illness spread or alleviate personal health burdens. However, the flexible nature of remote work might inadvertently reinforce the notion that employees must be visibly present even when seriously unwell [23]. This can negatively impact their well-being as they push themselves to work despite needing regular time off [17]. Working after hours contributes to adverse mental health outcomes such as occupational stress, and adverse physical health outcomes such as a higher risk of developing occupational injuries [24]. These negative impacts of working after hours can significantly impair productivity and work performance in the workplace [24]. The rapid change in working arrangements also contributes to work-related stress, which can be heightened by personal stressors associated with remote work [25], such as changing of tasks, homeschooling children while working [26], or caring for elderly family members [27]. Additionally, the pandemic itself can contribute to anxiety for individuals based on rising death tolls and a general fear of unforeseen circumstances [28]. As such, it is crucial to investigate presenteeism as the workforce navigates virtual environments and seeks to maintain a healthy work-life balance.

Remote work presents both advantages (e.g., work efficiency improvements, greater control of work tasks, better work-life balance [29]) and challenges (e.g., reduced social interaction, diminished work incentives, longer working hours, distractions at home, contributing to decreased productivity [30]). The sudden transition to remote work posed significant challenges for employees balancing household responsibilities and professional duties, further exacerbated by feelings of obligation and heightened presenteeism [31]. Evidence suggests that absenteeism is associated with employment dissatisfaction and reduced engagement in remote work environments [32]. The shift to telecommuting has led to decreased job engagement and attendance issues, exacerbated by psychiatric symptoms related to COVID-19 [28]. Employees in remote work environments may face increased stress and time restrictions, leading to burnout and loss of motivation [30].

This scoping review aims to examine the occurrence of presenteeism and absenteeism among remote workers during the COVID-19 pandemic and the interrelated physical and mental health issues during these periods. By identifying gaps in the literature, the review seeks to inform interventions aimed at safeguarding employee well-being in remote or hybrid work environments, with implications for both physical and mental health [31]. Our goal is to delve into the existing evidence on this topic and to identify areas where further research is needed before the conduction of a systematic review. The objectives of this review indicate that a scoping review is more advantageous as it will allow for the fulfillment of the required purposes. Munn et al., states that using a scoping review as a precursor to a systematic review is advantageous as it allows review teams to assess the scope of the literature before embarking on a more comprehensive review such as a systematic review [33]. Systematic reviews can subsequently be developed based on the initial evidence gathered during the scoping review stage. Therefore, we believe that a scoping review is the optimal approach to outline the current state of knowledge to pave the way for future research in this new and emerging field of research.

## Materials and methods

### Study design and registration

The review was designed using the Preferred Reporting Items for Systematic reviews and Meta-Analyses extension for Scoping Reviews (PRISMA-ScR) checklist [34]. No ethical approval was required to conduct this study at our institution.

### Search strategy

The literature search was conducted across multiple databases, including MEDLINE, PsycINFO, Embase, CINAHL, Eric, Business Source Premier, SCOPUS, and Sociological Abstracts. The preliminary search was conducted on Medline (see Table 1) and the databases were searched on November 21^st^, 2023. A secondary search of reference lists was also conducted.

**Table 1:** Sample search strategy employed on Medline.

Line #	Syntax
1	(Remote* OR {Remote work*} OR {telework*})
2	AND ({Occupational Stress} OR {Job Fatigue} OR {Psychological Stress} OR {Mental Health} OR {mental disorder} OR {mental illness} OR {Physical Fatigue} OR {Physical stress} OR {Emotional Exhaustion} OR {mental fatigue} OR stress OR fatigue OR burnout)
3	AND ({Absenteeism} OR {presenteeism})
4	AND ({COVID*} OR {pandemic*})

### Inclusion and exclusion criteria

To be eligible for inclusion in this scoping review, studies need to meet specific criteria, which include: (1) participants who were at least 18 years old; (2) employed in either full-time or part-time positions; (3) transitioned from in-person work to remote work due to the COVID-19 pandemic; (4) utilized experimental, quasi-experimental, prospective, and retrospective observational, case-control, analytical cross-sectional, or descriptive observational study designs; (5) encompassed randomized and non-randomized controlled trials; (6) presented in the English language; and (7) presenteeism and absenteeism concepts and their impact on worker health and productivity.

Studies were excluded if they: (1) included workers whose remote transition was voluntary and not related to the pandemic or workers who have history of remote work experience prior to the COVID-19 pandemic; (2) were review articles; (3) discussed non-work-related health issues or productivity factors.

The research team applied the population, concept, context (PCC) framework to categorize the inclusion and exclusion criteria (Table 2).

**Table 2:** Outline of PCC framework for the scoping review.

PCC Framework	Inclusion Criteria	Exclusion Criteria
Population	Adults aged 18 or older, engaged in full-time or part-time remote work	<18 years of age or 18+ but unemployed
Concept	Concepts of presenteeism and absenteeism and their impact on teleworkers’ health, efficiency and productivity	Exploring diverse factors affecting teleworkers’ performance and well-being beyond the scope of presenteeism and absenteeism
Context	Remote environments	In-person environments
	Research publications written in English	Non-English written publications

### Data collection

Two reviewers independently conducted a comprehensive literature screening to identify relevant studies based on the predefined inclusion and exclusion criteria. The screening process was facilitated using Covidence, which is a collaboration software for literature reviews [35]. This screening process consisted of several checkpoints, including the initial title and abstract screening, a mid-point review, and a final confirmation stage. At each checkpoint, the reviewers reconvened to discuss any discrepancies in their screening decisions and to refine the inclusion criteria as needed. This iterative approach helped ensure thorough and consistent screening across all identified studies. Following the title and abstract screening, a full-text review was conducted for the included studies that met the inclusion criteria. During this phase, reviewers carefully assessed the full texts to determine if the studies still met the inclusion criteria and did not violate any of the exclusion criteria. Any conflicts or uncertainties regarding study eligibility were discussed between the reviewers, and consensus was reached through mutual agreement or consultation with the first author (BN-K). Inter-rater reliability was assessed periodically throughout the screening and review process to gauge the level of agreement between the two reviewers. Measures such as the Cohen’s Kappa coefficient [36] were employed to quantify the agreement and ensure consistency in study selection. Kappa values ranging from 0.38 to 0.58 indicate moderate inter-rater reliability for full text screening, noting considerably high proportionate agreements (between 67–83%).

### Data extraction

Upon completion of the screening and review stages, data extraction commenced from the included studies. Two researchers (AMH, B-ZSL) independently extracted data from the papers included in the scoping review. This process involved capturing information on various aspects related to the study objectives, including target population demographics, methods employed, key findings concerning absenteeism, presenteeism, and their implications, as well as conclusions discussing the impact of transitioning to remote work on employee health. Specifically, the following categories were created and charted into Microsoft Excel [37]: (1) author(s) and date; (2) publication title; (3) publication; (4) aim of the study; (5) study setting; (6) sampling method; (7) study design; (8) data collection method(s); (9) data analysis; (10) conclusion; (11) outcomes; (12) most relevant findings; and (13) additional comments. Any conflicts or discrepancies encountered during the data extraction process were discussed and resolved with input from the senior research member (BN-K) to ensure accuracy and consistency in data extraction.

### Data synthesis

Data extraction was used to assess information on population demographics, methods, and key findings relevant to the implications of transitioning to remote work. Two independent reviewers (AMH, B-ZSL) collaboratively identified overarching themes they considered to provide the most comprehensive summary of the literature. Subsequently, these reviewers convened to engage in detailed discussions aimed at refining and finalizing the identified themes. Descriptive thematic analysis was performed using the Braun and Clarke (2006) framework to guide the development of themes [38]. This six-step process involved the researchers reviewing the data and noting down initial ideas for the codes. They then systematically coded significant items in the data set and collated data relevant to each code. Next, codes were grouped into potential themes, and all data relevant to each theme was gathered. The research team then verified the themes with the coded extracts and the entire data set. Additionally, the themes were further assessed and refined. This meticulous and inclusive approach ensured the validity and robustness of the topics and categories.

## Results

**Fig 1:**
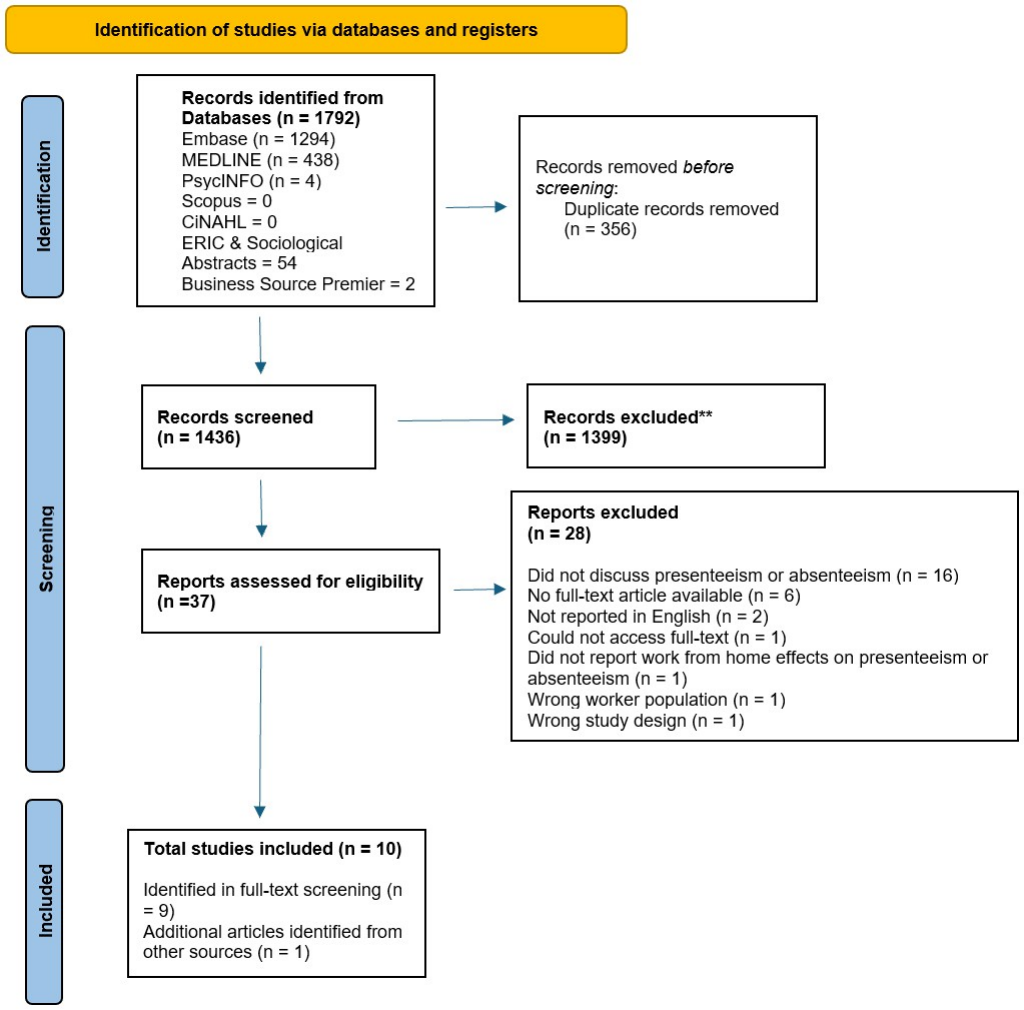
PRISMA flow chart outlining the screening process.

For this review, 1399 research papers underwent title and abstract screening. Subsequently, 1371 articles were excluded since they did not meet our inclusion criteria, leaving 28 articles for full text review. Upon completion of the full-text review, a total of nine studies were identified in Covidence to be included in our review. An additional reference, identified from the references of an article was included, leading to a total of 10 studies for data extraction. This process is outlined below in detail in Fig 1.

### Study characteristics

The ten included studies were conducted in various countries, including Canada [11, 39], the United States [40], Turkey [41], Portugal [42], Japan [43], the United Kingdom [28, 44], South Korea [45], and Malta [5]. Among these studies, seven cross-sectional studies [5, 11, 28, 39, 41, 42, 45] provided an overview of workforce experiences during the pandemic; two were qualitative studies [40, 44] delving into individuals’ perceptions and experiences in remote work environments; and one was an observational study [43] offering insights into the long-term consequences of remote work on worker productivity and well-being. Table 3 provides further details on the country, study population, and sample size.

**Table 3:** Study characteristics of studies analyzing presenteeism and absenteeism in remote work during the COVID-19 pandemic.

Author, Year	Country; Study Design	Study Population	Sample Size
Adisa et al., 2023 [44]	United Kingdom; Qualitative	Teleworkers	32
Chowhan et al., 2021 [39]	Canada; Cross-sectional	Employed Individuals	2653
Fiorini, 2023 [5]	Malta, Europe; Quantitative cross-sectional study	IT Teleworkers	459
Magalhães et al., 2022 [42]	Portugal; Cross-sectional	Non-academic university staff	322
Mullins et al., 2022 [40]	United States; Qualitative	Federal employees, federal contractors and others	1589
Parent-Lamarche and Laforce, 2022 [11]	Quebec, Canada; Cross-sectional	Health care workers, social workers, personal care attendants, occupational therapists, physiotherapists, health-related professionals (e.g., nutritionist, specialized educator, psychologist, laboratory technician, medical secretary).	1128
Ryoo et al., 2023 [45]	South Korea; Cross-sectional	White-collar wage employees	656
Şentürk et al., 2021 [41]	Turkey; Cross-sectional	Remote Workers	459
Shimura et al., 2021 [43]	Tokyo, Japan; Observational	Remote Office Workers from tertiary industries	3123
Van et al., 2020 [28]	United Kingdom; Cross-sectional	University staff and students	1055

**Fig 2:**
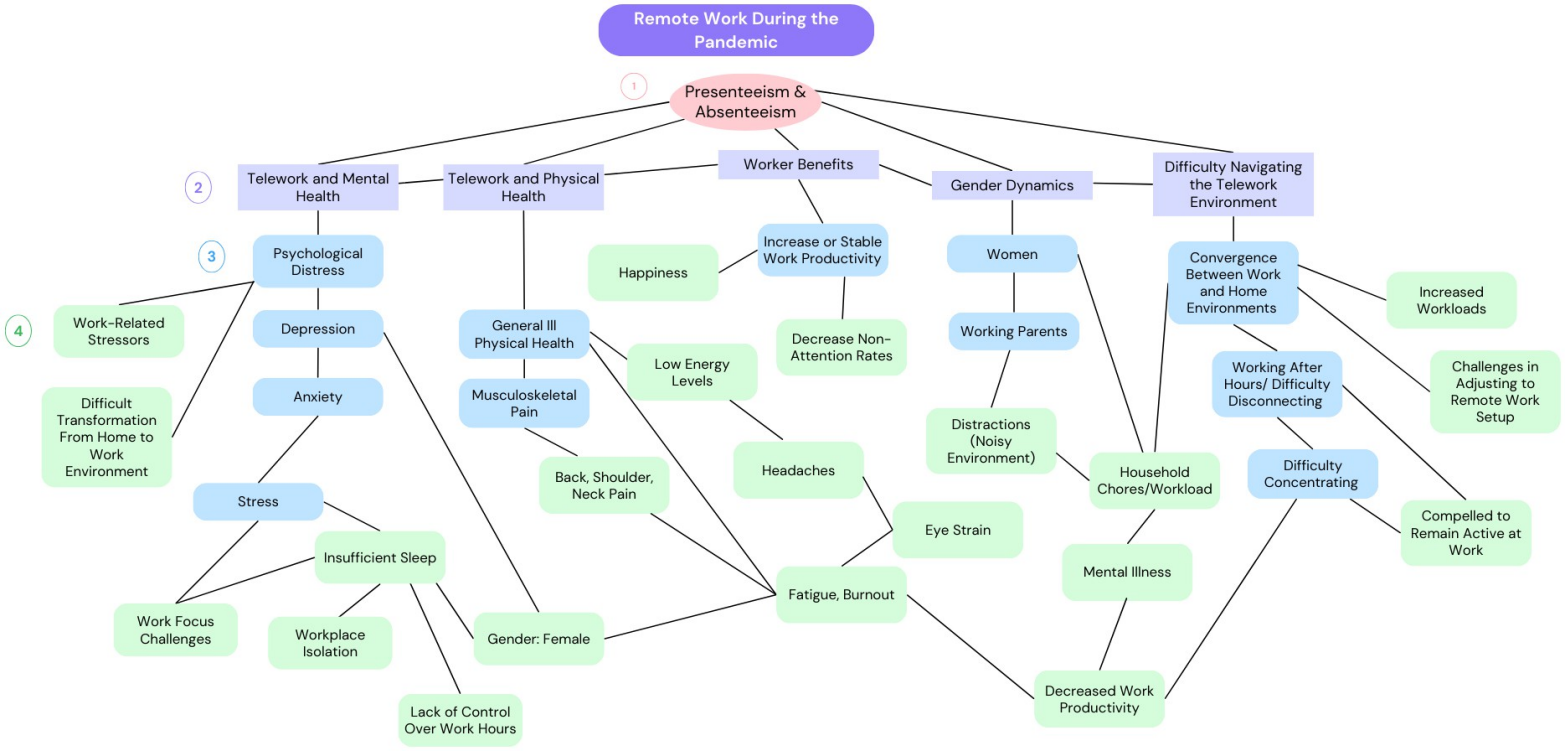
Concept map of identified themes of remote work during the pandemic associated with presenteeism and absenteeism.

Table 4 and Fig 2 provide a summary of the identified themes from the literature. The main overarching themes include telework and mental health, telework and physical health, worker benefits, gender dynamics, and navigating telework. Additionally, the table lists further sub-categories under each main theme, offering a more comprehensive perspective on the literature.

**Table 4:** Major themes regarding remote work during the pandemic within the 10 selected articles included in the full-text review.

Themes	Sub-Categories	Studies
Telework and Mental Health	Psychological Distress	The COVID-19 pandemic exacerbates work-related stressors, such as increased workload, contributes to psychological distress (β = 2.306, p ≤ 0.01) and absenteeism (β = 0.392, p ≤ 0.05) among university staff and students [11, 28].
		Difficulty in transforming the home into a conducive work environment hampers productivity and concentration, potentially causing psychological distress [44].
	Depression	Factors predicting depression comprised insufficient sleep (β = 0.378, p < 0.001), work-related focus challenges (β = 0.138, p < 0.001), female gender (β = 0.137, p = 0.001), workplace isolation (β = -0.133, p = 0.001), and lack of control over work hours (β = -0.094, p = 0.020), with poor sleep being identified as the primary predictor of depression [41].
		Depression levels are connected to both presenteeism and absenteeism (r = 0.435; and r = 0.133, respectively) [28].
	Anxiety	Remote work leads to increased anxiety [28, 41].
		Telecommuters are more likely to have anxiety (AOR = 2.82; 95% CI = 1.93–4.10) and insomnia (AOR = 1.93; 95% CI = 1.30–2.37) compared to in-person workers [45].
		Presenteeism is often accompanied by psychological symptoms, such as anxiety (36.1%) and stress (37.1%) [42].
	Stress	Telework is believed to relieve stress for some aspects in relation to work [40].
		Stress remains prevalent among remote workers (i.e., 80.4% experience normal stress, 19.4% mild stress, 0.2% moderate stress), with poor sleep quality (p < 0.001) and trouble focusing (p < 0.001) being predictors of stress [41].
		Stressed professionals, dealing with presenteeism—displaying physical symptoms like back pain (42.3%) and headache and/or migraine (36.1%)—showed reduced work performance, particularly among those managing both on-site and remote work (OR = 4.1; 95% CI = 1.8–9.6), compared to those primarily engaged in telework [42].
		Remote work is associated by some with reduced physical and psychological stress for some (p = 0.003) [43].
		The loneliness of remote working has a negative impact on stress reactions and work productivity ultimately impacting employee engagement [44].
		Stress levels, anxiety and depression, are correlated with those presenting presenteeism (r = 0.435) and absenteeism (r = 0.133) [28].
Telework and Physical Health	General Ill Physical Health	The heightened risk of anxiety and distress associated with sleep deprivation can exacerbate the challenges of coping with stress among remote workers, potentially leading to decreased energy levels [41].
		Physical symptoms such as back pain (42.3%) and headaches (36%) were the most commonly reported concern by teleworkers experiencing presenteeism. Physical symptoms were not statistically significant in their association with presenteeism (OR = 1.4 (0.2–10.1), p = 0.723) [42].
		Telecommuting is associated with a higher prevalence of insomnia (AOR = 1.93; 95% CI = 1.27–2.92), and other physical symptoms such as fatigue (AOR = 1.76; 95% CI = 1.30–2.37), and headache-eye strain (AOR = 1.94; 95% CI = 1.48–2.54) [45].
		Working outside of designated hours is linked to the onset of pain, increased occurrence of painful body areas, and the development of back (OR: 1.80; 95% CI: 1.15–2.84; p = .01), neck (OR: 2.07; 95% CI: 1.28–3.01; p = .002), and shoulder pain (OR: 2.17; 95% CI: 1.31–3.60; p = .003) [5].
	Musculoskeletal Pain	Telecommuting is associated with high MS pain (AOR = 1.76; 95% CI = 1.33–2.32) [45]. Whether MS pain was present had a strong correlation with burnout levels (OR = 1.46; 95% CI = 1.27–1.89; p < 0.001) [5].
		as well as the amount of exercise people engage in (OR = 0.83; 95% CI = 0.76–0.91; p < 0.001).
Worker Benefits	Increase or Stable Work Productivity	In the United States, telework was said to relieve stress, increase productivity, decrease non-attention rates and absenteeism, and make employees happier [40]. Further, telework was said to positively impact their work-life balance with those engaging in partial telework having no significant difference (p > 0.05) in work productivity compared to in-person work [43] therefore reducing presenteeism and absenteeism.
Gender Dynamics	Women	Females experienced increased household chores (p = 0.001) and workload (p = 0.012) along with many distractions (p = 0.009) impacting their work productivity [41]. Being female was significantly (p < 0.05) associated with mental illness, specifically depression and insomnia, a trend not observed for male teleworkers [45], as being female is linked with higher vulnerability to psychological distress and thus, presenteeism, and absenteeism [28].
	Working Parents	Women experienced more distractions for their children during telework than men [41].
		Full-time remote work increased stress reactions (p = 0.005) and decreased work productivity due to the noisy environment created by the workers children (p < 0.01) [43].
		Parenthood during pandemic-induced remote work is associated with psychological distress (β = −0.09), which is correlated with presenteeism (r = 0.435) and absenteeism (r = 0.133) [28].
Difficulty Navigating the Telework Environment	Convergence Between Home and Work Environments	The abrupt change from in-person to remote work due to COVID-19 resulted in increased workloads, online presenteeism, and challenges in adjusting to unfamiliar remote work setups [44].
		Individuals primarily engaged in telework often feel compelled to remain active at work even when they are unwell (i.e., presenteeism) [42].
		Women (β = 0.324, p < 0.001) engaged in telework experience an increase in household responsibilities (p = 0.001) leading to greater distractions during work (p = 0.009) [41].
		Struggling to disengage from work, individuals find it challenging to decipher between working and non-working hours (OR = 1.82; 95% CI = 1.02–2.56; p = 0.04), highlighting the intersection between work and the home environment [5].
	Working After Hours/ Difficulty Disconnecting	The rise in workload since COVID-19, accompanied by increased family duties, has led to decreased work engagement and a surge in "online presenteeism," contributing to longer work hours, never-ending virtual meetings, and pressure on employees to remain constantly connected virtually [44].
		The intricate relationship between working during nonworking hours, difficulty disconnecting, and the associated psychosocial risk factors among teleworkers leads to detrimental consequences such as physical pain and burnout (OR = 1.46; 95% CI = 1.27–1.89; p < 0.001) [5] and is therefore linked to increased presenteeism and absenteeism.
	Difficulty Concentrating	Turkish remote workers found it took longer to focus on tasks during telework and distractions (β = 0.165, p < 0.001) associated with this decreased productivity [41].
		Remote employees have higher task loads which leads to reduced work engagement and a sense of a constant need to have online presence (even outside of work hours) contributing to presenteeism [44]. These difficulties were exacerbated by the household disturbances further impacting concentration.

### Telework and mental health

#### Psychological distress

Three studies recognized the impact of telework on psychological distress and its relation to absenteeism and presenteeism during the pandemic. Notably, a higher correlation was observed between psychological distress and presenteeism (r = 0.435) compared to psychological distress and absenteeism (r = 0.133) [28]. During the COVID-19 pandemic, many teleworkers acknowledged an increased workload associated with working from home [11], which tended to lead to presenteeism as in order to keep up with the increased pressures, they continue to work even when ill. When workload increased, so did levels of psychological distress which is indirectly associated with higher rates of absenteeism (β = 1.511, p ≤ 0.01) [11]. However, recognition (i.e., intangible compensation through appreciation received from colleagues) was indirectly linked to lower instances of absenteeism via its impact on psychological distress (β = -0.881, p ≤ 0.001) [11]. Even though recognition was beneficial in mitigating psychological distress, it alone may not adequately support workers coping with telework-related stressors. Additionally, difficulties in adapting one’s home environment to a workspace could lead to psychological distress, affecting productivity and concentration (i.e., presenteeism) due to the poor ability to adjust [44]. Further, the diminishing interactions and interpersonal relationships among co-workers depletes the vital social and personal aspect of work, impacting employee engagement [44]. Van Der Feltz-Cornelis et al. [28] reported that 98% of their university staff worked from home experienced high levels of psychological distress, presenteeism, and absenteeism. Factors contributing to these vulnerabilities included being female, having children, being of younger age, having a somatic or functional syndrome, and self-isolation. Social isolation, lack of outdoor space, and low exercise levels were identified as contributors to presenteeism. Access to green spaces during telework was associated with lower presenteeism [28]. Before the pandemic, the average annual absenteeism rate was 11.6 days as of 2020; however, with the increase in telework by 2022, this average rose to 18.08 days [11].

#### Depression

One study correlated the presence of depressive symptoms to both presenteeism (r = 0.435) and absenteeism (r = 0.133) [28]. It is not uncommon for teleworkers to experience depression, as evidenced by a study in Turkey where 17.9% (i.e., 82 out of 459) of participants reported symptoms of depression. The challenges of working online in home environments, coupled with difficulties in focusing at home, can act as predictors of depression. This can subsequently reduce work productivity and performance [41] and lead to presenteeism and absenteeism as psychological well-being decreases.

#### Anxiety

In our review, four studies correlated anxiety due to online work with higher levels of presenteeism and absenteeism. A study on remote workers in Turkey revealed that 19.6% (i.e., 90 out of 459) of participants experienced anxiety. In a Portugal study, presenteeism was experienced by 30.1% (96 out of 322) of workers, many of whom experienced psychological symptoms of presenteeism [42]. In South Korea, teleworkers were likely to have anxiety than in-person workers, further revealing a significant association between presenteeism and work from home, with females being more susceptible to poor mental health outcomes [45]. Absenteeism, on the other hand, was not found to be associated with the anxiety of teleworking within this population. Within the UK, university staff experienced psychological problems such as anxiety impacting their presence at work during working hours [28]. Staff who showed resiliency to the negative impacts of the new work environments due to COVID-19 displayed fewer anxiety symptoms.

#### Stress

Six studies showcased the relationship between stress, presenteeism and absenteeism, particularly in the context of the COVID-19 pandemic, and the use of remote work arrangements. A study involving federal employees in the United States indicated that telework, often implemented in response to the pandemic, was perceived positively by employees in terms of stress relief and increased productivity due to reduced commute time [40]. Remote work has also been linked to a reduction in both psychological and physical stress responses (p = 0.003), independent of changes in job stressors and social support [43]. However, the transition to remote work has not been without challenges. Research indicates a notable prevalence of depression, anxiety, and stress among remote workers (i.e., 80.l4% experience normal stress, 19.4% mild stress, 0.2% moderate stress), with poor sleep quality (p < 0.001) and trouble focusing (p < 0.001) emerging as a significant predictor of these mental health issues [41]. Sleep deprivation, exacerbated by remote work conditions, not only elevates the risk of anxiety and distress but also diminishes employees’ capacity to cope with stress effectively. Moreover, the concept of presenteeism, wherein employees remain at work despite illness or reduced productivity, has been associated with physical symptoms such as back pain (42.3%) and headaches (36.1%), as well as psychological symptoms including anxiety (36.1%) and stress (37.1%) [42]. Remarkably, professionals who work both remotely and on-site report higher rates of presenteeism than those who work remotely most of the time (OR = 4.1; 95% CI = 1.8–9.6; p = 0.001). This implies that problems related to stress can worsen if the lines between work and home settings become blurred. A condition known as "online presenteeism" has resulted from the shift to remote work, which has also brought up new pressures such as an increased workload and the expectation to be always online [44]. This constant “connectedness” can drain important personal and social capital, which will lower employee engagement and increase stress levels. Van der Feltz-Cornelis et al. demonstrated a significant elevation in staff members’ stress levels due to the COVID-19 epidemic, further emphasizing the correlation between stress, presenteeism (r = 0.435) and absenteeism (r = 0.133) [28]. Excessive levels of stress, anxiety, and depression have been linked to both presenteeism and absenteeism, indicating a complicated relationship between mental health and work attendance habits.

### Telework and physical health

#### General poor physical health

The relationship between poor physical health, presenteeism and absenteeism has been outlined in four studies. Sleep deprivation, a significant contributor to anxiety and distress, poses a challenge for remote workers [42]. It reduces their energy levels, making it difficult for workers to cope with the demanding conditions of working from home [41]. The propensity for working during non-working hours has been linked to the development of pain in multiple body areas, including the back (OR = 1.80; 95% CI = 1.15–2.84; p = 0.01), neck (OR = 2.07; 95% CI = 1.28–3.01; p = 0.002), and shoulders (OR = 2.17; 95% CI = 1.31–3.60; p = 0.003), suggesting a correlation between extended work hours and physical discomfort [5]. Physical symptoms such as back pain (42.3%) and headaches (36%) have been identified among individuals experiencing presenteeism, further highlighting the detrimental effects of poor physical health on work performance [42]. Telecommuters are vulnerable to the adverse effects of ill physical health, as evidenced by higher rates of insomnia compared to in-person workers (AOR = 1.93; 95% CI = 1.27–2.92) [45]. Additionally, telecommuting has been significantly associated with fatigue (AOR = 1.76; 95% CI = 1.30–2.37) and head and eye strain (AOR = 1.94; 95% CI = 1.48–2.54), indicating a connection between remote work and deteriorating physical health [45]. As telework worsens physical health, higher rates of absenteeism and presenteeism are inevitable.

#### Musculoskeletal pain

According to a study in Portugal analyzing non-academic university staff, many employees work during telework while they are ill (i.e., presenteeism). Specifically, many workers are continuing to engage in telework while experiencing high levels of back pain (42.3%) [42]. A South Korean study also found that telecommuting was significantly associated with musculoskeletal (MS) pain (AOR = 1.76; 95% CI = 1.33–2.32) [45] a trend observed across various remote jobs in different countries. In Malta, 20% of participants reported pain in one area of their body, with an additional 19% experiencing pain in two body areas [5]. Back pain was the most profound issue reported (40%). The level of MS discomfort showed a strong correlation with the amount of exercise the participants engaged in (OR = 0.83; 95% CI = 0.76–0.91; p < 0.001), and the level of burnout they experienced (OR = 1.46; 95% CI = 1.27–1.89; p < 0.001) [5]. Those experiencing burnout reported more back (p = 0.04) and neck pain (p = 0.02), but not shoulder pain (p = 0.08). Half of the sample had experienced MS pain within the past 12 months of telework; however, there appears to be no significant difference in risk of back pain compared to those who work in person (OR = 1.15; 95% CI = 0.72–1.86; p = .58) [5].

### Worker benefits

#### Increased or stable work productivity

One study, based on federal employees in the United States, found that telework could relieve stress at work, increase work productivity, decrease non-attention rates and absenteeism, and contribute to employee satisfaction [40]. These employees perceive the pandemic-induced telework as positively impacting their work-life balance, primarily due to the time saved from daily commuting [5]. Other studies find that for partial remote work (i.e., less than 5 days a week), there is no significant difference (p > 0.05) in work productivity between in-person part-time work and online part-time work, making online work a viable option for this employee group [43].

### Gender dynamics

#### Women

A total of three studies addressed the experiences of women teleworking during the COVID-19 pandemic. Remote workers in Turkey who are women reported in a questionnaire that while teleworking, they spend more time doing household chores (p = 0.001) and face increased workloads (p = 0.012) [41]. Furthermore, they felt that online working causes frequent distractions (p = 0.009) due to external factors in their home environment. Difficulty focusing on tasks at work is a predictor of depression and stress, reducing work productivity [41] and can possibly lead to presenteeism. In fact, female workers are more susceptible to mental illness during telecommuting than their male counterparts in a sample in South Korea, with being female acting as a predictor of depression [45]. Within a university staff population in the United Kingdom, being of the female sex contributed to the vulnerability of experiencing psychological distress, presenteeism, and absenteeism [28].

#### Working parents

Three studies discussed the impact of parenthood on remote work during the COVID-19 pandemic. Women specifically reported experiencing more distractions from their children while working from home compared to men [41]. Full-time remote work (i.e., five days a week) was found to negatively affect presenteeism, as the presence of children and a noisy environment can increase stress (p = 0.005) and reduce productivity (p < 0.01) [43]. Having children while working from home during the pandemic is a known vulnerability of psychological distress (β = −0.09), presenteeism (r = 0.435), and absenteeism (r = 0.133) [28]. Overall, parents faced significant challenges due to pandemic-induced remote work, as they struggled to balance home and work responsibilities, leading to exhaustion [40]. They believed it brought on new stressors and concerns that were not present in traditional work settings. Those with limited access to technology also experienced reduced productivity due to work exhaustion, heavy workloads, and burnout, all of which were exacerbated by childcare simultaneously [40].

### Navigating telework

#### Convergence between work and home environments

Four studies have examined the convergence of work and home environments, which has highlighted several issues related to presenteeism. The COVID-19 pandemic has caused a fast shift from traditional in-person labour to online work, which has intensified work and given rise to the phenomena of online presenteeism [44]. Workers have found it difficult to adjust to the new norms of working from home, which has resulted in a lingering sense that they must always be present and available in the virtual workspace—even when they are ill [42]. The difficulties of separating work from home life blurs the lines between work and non-working hours, making it harder to draw defined boundaries between work and personal time [5]. A Turkish study emphasized how women (β = 0.324, p < 0.001) who work from home are more responsible for household duties (p = 0.001) and face diversions, which adds to the feeling that one is always engrossed in work-related activities [41]. These outside distractions (β = 0.165, p < 0.001), coupled with the stress of juggling family responsibilities, may encourage presenteeism as people try to make up for what they perceive to be decreased productivity. This can lead to overworking and, thus, further productivity loss due to burnout. This highlights the intricate relationship between work and home life in remote work arrangements, where domestic duties intrude into allocated work hours and may encourage presenteeism among remote workers [41].

#### Working after hours/ difficulty disconnecting

Two studies have found that the frequency of working after hours and finding it difficult to detach from work has increased, especially during the COVID-19 pandemic when work demands have increased due to more tasks, family responsibilities, and the shift to remote work [44]. This has led to a phenomenon known as "online presenteeism," when employees experience constant pressure to be accessible online, attend to work-related requests, and maintain a high level of activity outside of regular working hours [44]. This constant connectedness blurs the boundaries between work and personal life by extending work hours and creating a cycle of never-ending virtual meetings. Employees also feel pressured to check and respond to emails and messages outside of set work hours, resulting in poorer work performance and disengagement from work [44]. Moreover, studies linking this habit to other negative health issues like burnout and physical health problems highlight how difficult it is to disconnect from work during non-working hours [5]. In addition to negatively affecting mental health, this difficulty to step away from work has real-world physical repercussions, such as the emergence of pain and musculoskeletal conditions such shoulder, back, and neck pain (OR = 1.46; 95% CI = 1.27–1.89; p < 0.001) [5]. Ignoring these difficulties might lead to a vicious cycle of overwork and burnout, which would increase rates of absenteeism as workers struggle to meet the demands of an "always-on" workplace.

#### Difficulty concentrating

Three studies identified difficulties concentrating while working from home during the pandemic. In Turkey, 27.5% of remote workers (i.e., 126 out of 459) indicated that focusing on work tasks often took longer in telework than in person work, with 5.2% (i.e., 24 out of 459) indicating that tasks always took longer [41]. Further, 33.8% of workers (i.e., 155 out of 459) indicate that they experienced distractions (β = 0.165, p < 0.001) during telework, reducing their work productivity. In the United Kingdom, work from home employees admit to higher task loads, ultimately reducing their work engagement and leading to online presenteeism, where employees feel the need to always be present online and responding to work-related tasks even outside of work hours [44]. This can lead to presenteeism as one feels burnt out from the constant pressure, they feel to be available [44] and the constant disturbances that one’s household environment is susceptible to [43].

## Discussion

We identified five themes regarding the occurrence of presenteeism and absenteeism among remote workers during the COVID-19 pandemic and the interrelated physical and mental health issues during these periods. These themes include telework and mental health, telework and physical health, worker benefits, gender dynamics, and difficulties in navigating the telework environment. We recognize that there is no scientific consensus regarding the definition of presenteeism in the existing literature. In some cases, it is referred to illness resulting in presenteeism (i.e., working while ill), decreased productivity (e.g., difficulty remaining productive due to improper ergonomic arrangements, prolonged periods of inactivity) and disengagement (e.g., when a worker, usually women, become disengaged from their work because they must attend to their family/household tasks).

### Mental and physical health

In examining the relationship between telework and mental health, our analysis reveals a complex interplay where heightened psychological distress emerges as a pivotal determinant of presenteeism and absenteeism. Factors such as increased workload [11], social isolation [5], and challenges in adapting home environments into conducive workspaces [5, 41, 42, 44] contribute to elevated levels of psychological distress among teleworkers [11, 43]. While recognition from peers may offer some relief [11], the prevalence of depressive symptoms [28, 41, 45], anxiety [28, 41, 42, 45], and stress [28, 40–44] underscores the urgent need for proactive interventions to safeguard mental well-being in remote work settings. The increase in psychological distress poses a significant concern for organizations due to its negative impact on employee well-being [46] and productivity. In fact, depression and anxiety costs the global economy US$1 trillion (about $3,100 per person in the US) annually due to reduced productivity [47]. Existing literature also found that worsened mental health among teleworkers can lead to decreased morale, reduced job satisfaction, and increased absenteeism, ultimately affecting organizational performance and bottom-line results [48, 49]. Moreover, the prevalence of presenteeism, where employees continue to work despite illness or reduced productivity due to mental health issues, further compounds the financial burden on institutions [50]. Presenteeism not only reduces individual performance but also contributes to overwork and burnout, contributing to a cycle of decreased productivity [51] and heightened healthcare costs for organizations [52]. While some companies have implemented employee assistance programs (EAPs) and mental health resources [53], there remains a need for comprehensive strategies and proactive interventions to tackle the root causes of psychological distress to promote a more mentally healthy work environment [54]. Such initiatives may include mental health training, flexible work arrangements, and destigmatizing conversations around mental health in the workplace [55]. By prioritizing employee well-being and fostering a supportive organizational culture, businesses can mitigate the adverse effects of telework on mental health and enhance overall productivity and performance [56].

Regarding the relationship between teleworking and physical health, our research reveals a complex effect on workers’ general well-being. While several teleworkers have claimed their health improved due to longer periods of sleep time, as they no longer needed to spend extended periods commuting [5], this does not necessarily indicate that individuals have experienced an improvement in the quality of their sleep. According to Kohyama (2021), sleep quality is superior to sleep quantity, the real index of importance is how truly restful the sleep was and the lack of disturbance [57]. Therefore, even though sleep quantity may increase, we cannot assume it will lead to a positive impact on teleworkers. Specifically, sleep deprivation is a common problem among teleworkers, which is made worse by long workdays and a lack of clear boundaries between work and home life [41]. Furthermore, it is typical to report experiencing musculoskeletal pain, especially back pain, which is linked to prolonged periods of inactivity and improper ergonomic arrangements [2, 5]. These issues with physical health not only reduce productivity at work, but also highlight the need for wellness initiatives and ergonomic interventions to lessen the negative impact of telework on physical health.

### Gender dynamics

The telework experience is significantly shaped by gender dynamics, especially the disproportionate load that women who telecommute bear [28, 41, 45]. These findings are also highlighted in existing literature. Çoban (2022) reported that women experience greater workloads and diversions when juggling home duties, which can lead to elevated stress levels and mental health issues [58]. Working parents’ experiences highlight the difficulties they encounter in balancing the demands of remote work with childcare duties, underscoring the need for focused support systems to address the gendered aspects of telework [28, 41, 43, 58]. The difficulties associated with telework disproportionately affect women, who are under more strain to simultaneously manage childcare, household duties, and remote work obligations [41]. This imbalance not only makes women’s mental health issues and stress levels worse, but it also keeps gender disparities in the workplace alive [28, 41]. Individuals whose health improved during telework attributed this improve to more quality time with their partners, children, and pets, while those whose health regressed felt the opposite [5]. Organizations must put women who are teleworkers’ welfare first, both ethically and to respect the principles of equity, diversity, and inclusion (EDI) [59]. In addition to depressing worker morale and productivity, ignoring the demands of women in the workforce maintains structural hurdles that impede professional growth and economic empowerment. Workplaces can adopt procedures and policies that support work-life balance and cater to the requirements of working parents to foster a more inclusive workplace. Initiatives that can help working parents successfully balance their obligations at home and at work include flexible scheduling options, remote work allowances, and on-site childcare facilities [56], incorporating virtual babysitting or remote-friendly childcare programs. The difficulties faced by working parents can also be lessened by creating an inclusive and equitable work environment for all staff members by encouraging a culture of understanding and support where workers feel comfortable talking about their caregiving responsibilities and asking for necessary help. The literature suggests that ill health leads one to exhibit presenteeism, but it also suggests that presenteeism goes beyond simply working in ill health, specifically through its relation to gender. Organizations may foster a more welcoming and encouraging work environment that benefits both employees and the business by putting a priority on the well-being of female teleworkers and putting in place helpful policies for working parents.

### The struggles of navigating telework

Teleworkers face numerous obstacles because of the merging of their home and work settings, such as trouble focusing and trouble unplugging from work [5, 41, 44]. The widespread use of teleworking causes a blurring of the lines between work and personal life, which exacerbates mental and physical health problems and promotes a presenteeism culture [41, 42, 44]. Past studies have outlined several strategies that are designed to help teleworkers navigate the difficulties of combining work and home environments, promoting productivity while maintaining boundaries between personal and professional domains [60, 61]. These strategies include helping to create designated workspaces, setting up ergonomic workstations, and encouraging daily routines with consistent work hours and breaks [61]. For that reason, proactive steps to foster work-life balance and improve support systems for remote workers are vital in addressing these issues.

### Potential benefits of telework

Teleworker testimonials revealed that telework could be beneficial to their work productivity and create a happier work environment for them [40, 43]. The main reason for this insight is due to the cost-effectiveness cutting down time on their commute to work daily and is a more cost-effective alternative. These same results, however, do not ring true for all teleworkers, especially working parents who suffered greatly due to teleworking [40]. The mixed success rates of working from are due to factors such as the lack of social integration the job offers its employees and the lack of supervisor support and organizational trust that they feel during work from home [40]. These types of strains make it difficult for workers to connect with their co-workers properly and this can lead to further health concerns which will indirectly impact other areas of work.

### Limitations

Our study is limited in the fact that we only included studies that were written or translated into English language studies countries, potentially overlooking valuable insights from non-English language studies in other countries. Future reviews may contemplate the incorporation of studies published in languages other than English. Another limitation of this scoping review is the exclusion of relevant information from grey literature sources, such as unpublished reports, both published and unpublished conference proceedings, or organizational documents, which could have provided valuable insights into the occupational consequences associated with the transition to remote work during the COVID-19 pandemic. Not only that, only one study discussed how the frequency of telework (full time vs part time) could affect worker productivity and contribute to presenteeism and absenteeism in work from home settings. More research needs to be conducted to provide a more thorough analysis of the effect of teleworking on mental health in terms of work style.

### Future research and implications

The findings of this review lead us to conclude that to prevent negative effects on occupational health, it is critical that future studies explore more aspects of what can facilitate the shift from in-person to remote work. By doing this, improvements in office procedures that enable employees to flourish in their homes would be facilitated. Furthermore, additional research must be done to determine what kinds of resources (e.g., psychosocial support) teleworkers require from their employers and how to provide them to the teleworking community in a stigma-free manner. Following the COVID-19 pandemic, occupational health will be able to positively impact telework if stakeholders can comprehend how to support workers’ transition to telework and can assemble helpful materials to aid them. Future research should prioritize the implementation of longitudinal studies extending over 5 to 10 years. Such an extended timeframe would allow for a thorough exploration of the nuanced dynamics surrounding presenteeism and absenteeism within the context of remote work arrangements. Moreover, qualitative investigations that explore the complex realities and nuances of distant work environments are needed. These qualitative studies could provide vital insights into the various opportunities and problems people have when navigating remote work situations, which would further our understanding of the effects of these environments on professional productivity and well-being. Additionally, there seems to be a lack of consensus within the literature in relation to the definition of presenteeism (e.g., working while ill, loss of productivity due to poor ergonomics, being disengaged due to resorting to other tasks separate from work during working hours). For this reason, we recommend future research attempts to reach consensus and develop a singular definition of presenteeism to be used across literature.

To foster a greater understanding of the underlying mechanisms and theoretical frameworks that support the structure of remote labor, future research initiatives should also shift their attention towards theory generation. Researchers can provide a more comprehensive interpretation of the complexities present in modern work arrangements by shedding light on the complex interactions between different elements like organizational culture, technological affordances, and personal preferences by exploring the theoretical perspectives influencing the phenomenon of remote work.

This scoping study emphasizes the need for all-encompassing policies and interventions which promote the physical and mental health of remote workers. We must acknowledge that presenteeism is an underdeveloped concept with new complexities of its definition arising as teleworking becomes more prominent due to the recent pandemic. We recommend employers to prioritize the creation of stress reduction strategies, work-life balance initiatives, and ergonomic support services to avert physical health problems specific to telework by using a comprehensive approach accounting for the various intersecting health and wellness factors.

Extrapolating from our findings, it is important to consider strategies for monitoring and mitigating presenteeism and absenteeism in remote work settings. Organizations can develop and implement a portal where employees can report their presenteeism symptoms, such as the reporting of physical or mental health problems, the degree of impairment their illness creates at work, and productivity levels in recent time. Such a system can help monitor and mitigate the impact of early presenteeism on organizational productivity through early detection before repetitive and problematic presenteeism behavior occurs. A similar process can be created for the early detection of absenteeism. These enhance worker productivity and well-being and organizational efficiency through early detection of presenteeism symptoms. Future collaborative research is encouraged to support employers in co-creating survey questions and a portal tool that is in line with best available methods of measuring presenteeism, while being suitable to employers’ needs.

## Conclusion

The results highlight the complex effects of remote work arrangements on worker productivity and well-being, especially given the COVID-19 pandemic. Firstly, teleworking has advantages and disadvantages for mental health. Some people may benefit from shorter commutes and more flexible work schedules by feeling less stressed and more productive, but others may endure higher levels of psychological distress, anxiety, despair, and stress. Additionally, the long work hours and ergonomic difficulties that come with remote work situations can make telecommuters more prone to a variety of medical illnesses, such as musculoskeletal discomfort which may result in presenteeism and absenteeism. Further, gender dynamics are a major factor in determining telework experiences, as working parents and women encounter difficulties regarding childcare, domestic obligations, and workload. The significance of gender-sensitive policies and support systems in addressing the unique requirements of these populations in remote work environments is highlighted by these inequalities.

## Supporting information

S1 Table Quantitative study results. (DOCX)

S2 Table Gaps and limitations. (DOCX)

S1 File PRISMA-ScR checklist. (PDF)
