# Supplementary material for: Remote work transition amidst COVID-19: Impacts on presenteeism, absenteeism, and worker well-being—A scoping review
Source: PLoS One. 2024 Jul 18;19(7):e0307087. doi: 10.1371/journal.pone.0307087 (PMC11257327; doi:10.1371/journal.pone.0307087)
Supplement: S1 Table — (DOCX) [file pone.0307087.s001.docx]

| **Study Reference** | **Outcome Type** | **Outcome Measured** | **Statistic** | **Result** | **Confidence Interval** | **p-value** |
| --- | --- | --- | --- | --- | --- | --- |
| Chowhan et al., 2021 [39] | Absenteeism | Females Absence compared to Males | AOR | 2.67 | N/A | <0.01 |
|  |  | Poor health associated with (Age 65-74) | AOR | 3.238 | N/A | 0.002 |
|  |  | Poor Health | AOR | 11.752 | N/A | 0.012 |
| Fiorini et al., 2023 [5] | General Health | Change in General Health from Proportion of Remote Work | N/A | N/A | N/A | 0.26 |
|  |  | Change in General Health from Change in Remote Work | N/A | N/A | N/A | 0.014 |
| Magalhaes et al., 2022 [42] | Presenteeism | Productivity loss due to Presenteeism from hybrid work* compared to full remote or full in-person work | N/A | N/A | N/A | 0.001 |
|  |  | Workplace | N/A | N/A | N/A | 0.002 |
|  |  | Association Between Presenteeism and Sex | N/A | N/A | N/A | 0.362 |
| Parent-Lamarche and Laforce., 2022 [11] | Absenteeism | Psychological Distress | Pearsons Correlation | *r* = 0.24 | N/A | ≤0.01 |
|  |  | Recognition at Work | Pearsons Correlation | *r* = -0.16 | N/A | ≤0.01 |
|  |  | `Teleworking | Pearsons Correlation | *r* = -0.07 | N/A | ≤0.05 |
| Ryoo et al., 2023 [45] | Presenteeism | Telecommuting Impact | AOR | 1.66 | 1.20-2.28 | N/A |
|  |  | Increased Anxiety | AOR | 2.82 | 1.93–4.10 | N/A |
| Senturk et al., 2021 [41] | General Health | Work and Household Chores | N/A | N/A | N/A | 0.006, 0.012 |
|  |  | Workplace Loneliness | β | -0.094 | -1.071 - -0.283 | 0.001 |
|  |  | Stress |  |  |  |  |
| Shimura et al., 2021 [43] | Presenteeism | Job Stress | Pearsons Correlation | *r* = 0.405 | N/A | N/A |
|  |  | Sleep Disturbance | Pearsons Correlation | *r* = 0.387 | N/A | N/A |
| Van et al., 2020 [28] | Presenteeism | Psychological Distress | Pearsons Correlation | *r* = 0.435 | N/A | N/A |
|  | Absenteeism | Psychological Distress | Pearsons Correlation | *r* = 0.133 | N/A | N/A |
