# Supplementary material for: Remote work transition amidst COVID-19: Impacts on presenteeism, absenteeism, and worker well-being—A scoping review
Source: PLoS One. 2024 Jul 18;19(7):e0307087. doi: 10.1371/journal.pone.0307087 (PMC11257327; doi:10.1371/journal.pone.0307087)
Supplement: S2 Table — (DOCX) [file pone.0307087.s002.docx]

| Author | Gaps and Limitations |
| --- | --- |
| Parent-Lamarche and Laforce, 2022 [11] | 1. Non-representative sample  - 92.38% women  2. Cross-sectional, cannot confirm causality |
| Mullins et al., 2022 [40] | 1. Limited population  2. Lack of personal information given, made it difficult to merge perspectives and narratives among similar groups |
| Fiorini, 2023 [5] | 1. Cross-sectional, cannot confirm causality  2. Limited to only the IT sector |
| Adisa et al., 2023 [44] | 1. Small sample size |
| Senturk et al., 2021 [41] | 1. Cross-sectional, cannot confirm causality  2. Snowball sampling, limited representativeness, potential bias, high uniformity |
| Magalhaes et al., 2022 [42] | 1. Limited to only 1 university population, lack of generalizability  2. Cross-sectional, cannot confirm causality |
| Shimura et al., 2021 [43] | 1. Only tertiary industries in a limited regional area, lack of generalizability  2. Observational study of only 2 years  3. Lack of assessment of factors that substantially impact stress reactions and presenteeism  4. High participant drop our rate |
| Ryoo et al., 2023 [45] | 1. Cross-sectional, cannot confirm causality  2. Lack of survey items to assess confounding variables such as lifestyle, medical history, etc. |
| Chowhan et al., 2021 [39] | 1. Cross-sectional, cannot confirm causality  2. Did not assess employers only assessed employees, results in loss of relevant contextual information to provide operation and industry information |
| Van et al., 2020 [28] | 1. Cross-sectional, cannot confirm causality |
